# Protocol and research program of the European registry and biobank for interstitial lung diseases (eurILDreg)

**DOI:** 10.1186/s12890-024-03389-9

**Published:** 2024-11-18

**Authors:** Ekaterina Krauss, Silke Tello, Jennifer Naumann, Sandra Wobisch, Clemens Ruppert, Stefan Kuhn, Poornima Mahavadi, Raphael W. Majeed, Philippe Bonniaud, Maria Molina-Molina, Athol Wells, Nik Hirani, Carlo Vancheri, Simon Walsh, Matthias Griese, Bruno Crestani, Andreas Guenther, on behalf of further eurILDreg investigators, Raphael Borie, Caroline Kannengiesser, Venerino Poletti, Juergen Behr, Fotios Drakopanagiotakis, Helder Bastos, Claudia Ravaglia, Killian Hurley, RARE-ILD investigators, Roland Eils, Ivo Gut, Hossam Haick, Yoav Y. Broza

**Affiliations:** 1 European IPF/ILD Registry & Biobank (eurIPFreg/Bank, eurILDreg/Bank), Giessen, Germany; 2 Center for Interstitial and Rare Lung Diseases, Universities of Giessen and Marburg Lung Center (UGMLC), Justus-Liebig-University Giessen, Member of the German Center for Lung Research (DZL), Giessen, Germany; 3 Service de Pneumologie Et Soins Intensifs Respiratoire, Centre de Référence Constitutif Des Maladies Pulmonaires Rares de L’Adultes de Dijon, Centre Hospitalier Universitaire de Dijon-Bourgogne, INSERM U1231, Equipe HSP-Pathies, Faculty of Medicine and Pharmacy, Université de Bourgogne, Dijon, France; 4 ILD Unit, Respiratory Department, University Hospital of Bellvitge (HUB), Biomedical Research Institute of Bellvitge (IDIBELL), Barcelona, Spain; 5 Royal Brompton Hospital London, London, UK; 6 Centre for Inflammation Research, University of Edinburgh, Edinburgh, UK; 7 Department of Clinical and Experimental Medicine, Regional Referral Center for Rare Lung Diseases, University Hospital Policlinico, University of Catania, Catania, Italy; 8 King’s College Hospital Foundation Trust, Denmark Hill, London, UK; 9 ChILD-EU, Hauner Children’s Hospital, University of Munich, Member of the German Center for Lung Research (DZL), Munich, Germany; 10 Institute National de La Sainté Et de La Recherche Médicale, Hopital Bichat, Service de Pneumologie, Paris, France; 11 Cardio-Pulmonary Institute (CPI), Klinikstr. 33, 35392 Giessen, Germany; 12 Agaplesion Lung Clinic „Evangelisches Krankenhaus Mittelhessen“, Paul-Zipp Str. 171, 35398 Giessen, Germany; 13 Institute for Lung Health (ILH), Giessen, Germany; 14 eurILDreg Investigators, European ILD Registry (eurILDreg), Klinikstrasse 36, Giessen, 35392 Germany

**Keywords:** Interstitial Lung Disease, Idiopathic Pulmonary Fibrosis, European Registry and Biobank for Interstitial Lung Diseases, eurILDreg, eurIPFreg

## Abstract

**Background and Aims:**

Interstitial lung diseases (ILDs), encompassing both pediatric and adult cases, present a diverse spectrum of chronic conditions with variable prognosis. Despite limited therapeutic options beyond antifibrotic drugs and immunosuppressants, accurate diagnosis is challenging, often necessitating invasive procedures that may not be feasible for certain patients.

Drawn against this background, experts across pediatric and adult ILD fields have joined forces in the RARE-ILD initiative to pioneer novel non-invasive diagnostic algorithms and biomarkers. Collaborating with the RARE-ILD consortium, the eurILDreg aims to comprehensively describe different ILDs, analyze genetically defined forms across age groups, create innovative diagnostic and therapeutic biomarkers, and employ artificial intelligence for data analysis.

**Methods:**

The foundation of eurILDreg is built on a comprehensive parameter list developed and adopted by clinical experts, encompassing over 1,800 distinct parameters related to patient history, clinical examinations, diagnosis, lung function and biospecimen collection. This robust dataset is further enriched with daily assessments captured through the patientMpower app, including handheld spirometry and exercise tests, conducted on approximately 350 patients over the course of a year. This approach involves app-based daily assessments of quality of life, symptom tracking, handheld spirometry, saturation measurement, and the 1-min sit-to-stand test (1-STST). Additionally, pediatric data from the ChILD-EU registry will be integrated into the RARE-ILD Data Warehouse, with the ultimate goal of including a total of 4.000 ILD patients and over 100.000 biospecimen.

**Discussion:**

The collaborative efforts within the consortium are poised to streamline research endeavors significantly, promising to advance patient-centered care, foster innovation, and shape the future landscape of interstitial lung disease research and healthcare practices.

**Trial Registration:**

EurILDreg is registered in the German Clinical Trials Register (DRKS 00028968, 26.07.2022), and eurIPFreg is registered in ClinicalTrials.gov (NCT02951416).

## Background

Interstitial lung diseases (ILDs), pediatric and adult, spontaneous and familial, constitute a diverse range of chronic lung conditions with variable progression and prognosis: some are potentially reversible or can be stabilized. The remaining subtypes, unfortunately, frequently show a progressive pulmonary fibrosis (PPF) phenotype and, ultimately, a fatal course, similar to the Idiopathic Pulmonary Fibrosis (IPF) [1–5]. Apart from two novel antifibrotic drugs, and steroids/immunosuppressants used for inflammatory driven ILDs, therapeutic options are scarce and lung transplantation represents the only curative option [6, 7].

Data with regard to the natural course of ILDs and factors driving disease initiation and progression are limited and are mostly confined to IPF, one of the more frequent and aggressive forms of ILD [2]. Differential diagnosis of ILDs can also be very difficult and – up to now – often requires invasive procedures such as bronchoscopy with bronchoalveolar lavage and transbronchial cryobiopsy, or even video assisted surgical lung biopsy [8, 9]. In many cases, a correct diagnosis cannot be made with great confidence, because either the diagnostic results are inconclusive or patients are too old, comorbid or sick to undergo invasive procedures [10, 11].

Research on biomarkers has been conducted using peripheral blood, bronchoalveolar lavage fluid (BALF), and exhaled breath condensate (EBC) [12]. Numerous molecules involved in alveolar epithelial cell injury, proliferation and matrix remodeling as well as immune regulation have been proposed as potential biomarkers [13]. For instance, in prior investigations on Idiopathic Pulmonary Fibrosis (IPF), epithelial proteins like KL-6, Surfactant protein (SP)-A, SP-D, and other factors, such as those indicating oxidative stress (e.g., serum hydroperoxide), have been proposed as biomarkers capable of distinguishing between stable and progressive disease (e.g., CCL18 in serum) or indicating response to therapy (e.g., Toll interacting protein) [14–17]. Similarly, biomarkers derived from tissue samples, including the quantity of fibroblast foci, the expression level of Ki-67 (a marker of tissue proliferation), and caspase-3 (a marker of tissue apoptosis), were identified to carry prognostic implication [18].

Although, biomarkers have the potential to be highly useful for monitoring disease activity and assessing the therapeutic efficacy of treatments, there is currently no established biomarker in routine use for diagnosis and assessment of prognosis in ILD, and this holds particularly true for non-invasive markers [19]. Classifying biomarkers in the context of ILDs, especially IPF, poses challenges due to incomplete understanding of their functions, with ongoing studies progressing in this area. Despite this, biomarkers that prove valuable in managing IPF can be grouped into eight classes based on a clinical/physiopathological perspective. These include serum biomarkers (KL-6, SP-D, MMP-7), bronchoalveolar lavage fluid biomarkers (SP-D, KL-6, MMP-7), exhaled breath condensate biomarkers (hydrogen peroxide, nitric oxide, leukotrienes), lung tissue biomarkers (studied for IPF diagnosis but requiring invasive procedures), genetic biomarkers (such as MUC5B, telomere length), proteomic biomarkers (SAA, AAT), metabolomic biomarkers (sphingolipids, glycerophospholipids), and microRNA biomarkers (miR-21, miR-155). These biomarkers demonstrate utility in ILD (especially IPF) diagnosis, but limitations such as lack of specificity or reliance on specialized detection equipment constrain their diagnostic accuracy [20].

Further of interest are electronic noses (eNoses), which are artificial sensor systems, usually consisting of a range of sensors for various chemicals of interest; being able to detect patterns of volatile organic compounds (VOC) in different body fluids such as urine, blood, skin secretions, and exhaled breath and then use pre-established algorithms for classification of the ‘breath print’ for comparison with previously recorded samples [21, 22]. The concept of the eNose is that metabolic and biochemical processes occurring in different diseases give rise to specific patterns of endogenous VOC, resulting in a “volatolome” or a VOC signature which could be evaluated by eNose’s chemical sensors, and serve as possible markers of some inflammatory, microbial, oxidative and neoplastic conditions [23].

Implementing patient-centered care in ILD faces challenges in times when healthcare professional resources are getting increasingly scarce [24]. On the basis of digital innovations, the E-Health technology, such as home monitoring, offers promising options by enabling remote data exchange, facilitating frequent monitoring, and potentially allowing for earlier detection and intervention in ILD progression [25]. Therefore, home monitoring, particularly using handheld spirometry, has gained attention for its feasibility, reliability, and sensitivity in predicting disease progression and mortality in pulmonary fibrosis [26].

## Methods

Leading European ILD experts have joined forces to a) implement a novel, academically driven and completely independent registry and biobank for all forms of ILDs, the European Registry and Biobank for Interstitial Lung Diseases (eurILDreg and eurILDbank), b) establish a thorough and most suitable parameter set employed for deep phenotyping of these patients, and c) define short- to midterm research goals in these patients in the frame of the RARE-ILD (Raising Diagnostic Accuracy and Therapeutic Perspectives with the European Registry for ILD) consortium funded by the European Joint Program on Rare Diseases. For such purpose, a broad informed consent (IC) and a data protection concept meeting all ethical, legal and social issues, in particular requirements of the GDPR, needed to be developed.

In addition, software solutions for data capture and an overarching Data Warehouse (DWH) structure needed to be developed and to be compatible with the GDPR and other regulatory conditions. Finally, a work program needed to be established and to be implemented. Ultimately, the eurILDreg aims to include around 4000 ILD patients and over 100.000 biospecimen.

The parameter set was developed on the basis of existing pediatric (ChILD-EU) and adult (eurIPFreg) registries/biobanks [27, 28]. These initiatives, including ChILD-EU (http://www.klinikum.uni-muenchen.de/Child-EU/en/child-eu-register) and eurIPFreg (www.pulmonary-fibrosis.net), were established with funding from the European Commission's Frame Program 7 (FP7), and they evolved from the European IPF Network funded by the European Commission from 2008–2011. However, as several other conditions needed to be taken into account, major changes were introduced and agreed on in multidisciplinary video conferences. Likewise, the list of biospecimen reflected previous activities in the eurIPFreg, but was substantially expanded and was agreed on in an interdisciplinary fashion.

### Study inclusion and exclusion criteria

Consecutive, preferentially incident, adult and pediatric patients with all types of ILDs will be included in the registry without any selection bias, encompassing those with a formal diagnosis of a specific ILD or an unclassifiable ILD, who have signed IC, as they present at the sites. Given the broad spectrum of more than one hundred different forms of ILD, ranging from more common to extremely rare conditions, the registry will include IPF, chronic hypersensitivity pneumonitis (chron. HP), ILDs associated with collagen/vascular diseases (CVD-ILD), unclassifiable ILDs, cryptogenic organizing pneumonia (COP), desquamative interstitial pneumonia (DIP)/Respiratory Bronchiolitis–ILD (RB-ILD), fibrotic Non-Specific Interstitial Pneumonia (fNSIP), extremely rare ILDs such as Lymphangioleiomyomatosis (LAM), Hermansky-Pudlak Syndrome Interstitial Pneumonitis, progressive fibrotic sarcoidosis, drug-induced ILD, pleuropulmonary asbestosis, familial ILD (mostly familial idiopathic interstitial pneumonia [IIP] or IPF), and Pleuropulmonary Fibroelastosis (PPFE).

Patients will be included into the eurILDreg once they provided written informed consent. Cases, in which a final diagnosis cannot be reached with high certainty despite a multidisciplinary discussion, can be recruited as unclassifiable ILD (uILD), although biopsy should be taken whenever possible in such cases, to improve accuracy of diagnosis. Reflecting the character as „real world data registry “, there are no formal exclusion criteria.

### Ethics and informed consent

The eurILDreg informed consent is based on a template as recommended by the Permanent Working Party of the German Medical Ethics Committees (AKEK). It allows collection and long-term storage of human biospecimen (blood, BALF, breath condensates as well as tissue samples) together with related medical data to be used for a wide range of medical research purposes in the interest of maximizing the benefit to the general public (broad medical use), including carrying out genetic analyses and gene expression studies on blood samples (incl. whole genome analysis). It also allows passing on the biospecimen to both academic and industrial third parties. Measures for the protection of personal data are implemented in full accordance with GPDR and other regulations.

The IC has already been approved by the ethic committees of Giessen (AZ:111/08), Barcelona, Paris (0911932), Dijon (1504), Edinburgh, Catania (348). Additionally, approval from the medical ethics committee at each participating site will be obtained prior to patient inclusion. The informed consent from the patients is obtained by the respective local centers / data providers using paper based consent forms. Graded consent with individual limitations made by the patient is entered into the EDC system together with the date of consent. The original consent form remains with the data provider and is archived.

### Data entry platform

The eurILDreg operates on the REDCap platform, a secure and user-friendly web application designed for electronic data capture in research studies. The registry is configured to meet the specific needs of the data collection process for ILD research. Additionally, it is linked to an i2b2-based RARE-ILD DWH, enhancing its capabilities for comprehensive data management and analysis. The integration with i2b2, a known informatics framework for clinical research, provides a robust infrastructure for storing and managing diverse datasets collected within the eurILDreg. This linkage facilitates efficient data retrieval and analysis, enabling to conduct advanced queries across the ILD data. The combination of REDCap and i2b2 contributes to the overall functionality and effectiveness of eurILDreg as a comprehensive registry for ILD research.

All software systems operate within the certified computing center of the University Hospital Giessen and Marburg GmbH, ensuring data security through encrypted transfers and adherence to German IT security standards (IT Security act IT-SiG). Strict precautions, such as emergency power supply and redundant network links, further safeguard the physical hardware, reducing vulnerabilities. Transfers of data between all parties are strictly encrypted using transport layer security (TLS 1.2 +). No personal data or pseudonyms are transmitted via insecure internet connections or other media at any point. The data protection concept has been approved by JLU Data Protection Officer (V21/20).

### Central biobanking

The eurILDreg extends beyond data collection and includes a biobanking, with a harmonized Standard Operating Procedure (SOP)-based sampling algorithm and centralized storage of collected specimen. This involves the utilization of 2D-barcoded sample cups, ensuring digital management of biological samples with accurate and traceable identification. The biobank is further equipped with a robotic sample store (Liconic BiOLiX STC Kiwi 33k0 ULT, Liconic instruments, Mauren, Liechtenstein), which is capable of handling up to 2 million samples.

This technique streamlines the storage and retrieval processes, ensuring efficiency in managing a large amount of biological specimens. Tissue specimen and isolated cells are stored in 2D-barcoded tubes in the gas phase of liquid nitrogen in Chart MVE HEco 1800–190 tanks with a capacity of up to 60.000 aliquots.

Additionally, Rabbitfish (Informeleon LifeScience Technologies, Woerrstadt, Germany) is employed as a Biobank Information and Management System (BIMS), serving as an interconnected system with the overarching data management infrastructure (DWH). It plays a crucial role in the assessment of sample-related data (time stamps, sample quality, etc.) and tracking and managing of samples, providing a comprehensive solution for the organization and accessibility of biobanking data within the eurILD registry (Table 1).

**Table 1:** Biospecimen collection in the eurILDreg

	EDTA Blood 8 × 200 µl	Plasma 8 × 200 µl	Serum 8 × 200 µl	Urine 8 × 1 ml	EBC ~ 1-2 ml	BALF 16 × 1 ml	Brushing	Tissue
Baseline	x	x	x	x	x	x	x	x
Follow-up (every 3–6 months)	x	x	x	x	x	x	x	X
						If possible	If possible	If possible

### Deep phenotyping and data integration

In the eurILDreg enrollment procedure, individuals presenting with ILD symptoms such as dyspnea, cough, and loss of exercise capacity will be enrolled in the eurILDreg upon receiving an ILD diagnosis. The enrollment process involves a comprehensive deep phenotyping approach to thoroughly characterize the patients. Pediatric patient data will be obtained from the ChILD-EU registry and later integrated with eurILDreg data in the RARE-ILD DWH.

Deep phenotyping tool (published at 10.21961/mdm:46015.) not only includes the usual clinical routine data such as pulmonary function tests or gas exchange captured at the site, but also the administration of ILD- and Health-Related Quality of Life (HRQol) questionnaires, as well as a variety of clinical assessments, e.g. frailty assessment, exercise tests, laboratory values analysis, high-resolution computed tomography (HRCT) scans, handheld spirometry for pulmonary function, saturation monitoring, and accelometry for activity tracking [29].

In addition, biobanking activites centered on blood and bronchoalveolar lavages will be enriched by signatures of volatile organic solvents in exhaled breath (captured via awarded Sniffphone), of proteome and transcriptome in exhaled breath condensates (EBC), of the whole genome in blood cells and more complex 3D tissue cultures based on explanted lung tissue (precision cut lung slices obtained during lung transplantation).

Simultaneously, a multi-level omics approach will be implemented, delving into various biological layers to enhance the understanding of ILD. This involves the analysis of blood samples to explore the genome and epigenome, and the examination of exhaled breath condensates to investigate the proteome, transcriptome, and volatolome. These advanced omics analyses aim to provide a comprehensive molecular perspective, shedding light on the genetic, epigenetic, and molecular factors associated with ILD. The integration of clinical assessments and multi-level omics data is designed to offer in-depth understanding of ILD, contributing to development of personalized treatment.

In collaboration with Professor Simon Walsh and based on a National Institute of Health Research (NIHR) grant, imaging data are going to be analyzed by use of artificial intelligence. Ultimately, a big data set on ILDs will be pulled together in the RARE-ILD DHW, including all clinical, translational and basic data, and used to answer the above mentioned research questions.

The data generated from the comprehensive assessments and omics analyses will also be stored in the RARE-ILD DWH. This repository serves as a centralized hub for the integration of diverse data types, facilitating advanced analyses and insights through big data methodologies. The integrated data will undergo sophisticated analysis techniques, including unsupervised clustering facilitated by artificial intelligence. This approach aims to identify distinct patient subgroups based on shared characteristics, enabling a more nuanced understanding of the heterogeneity within the ILD population.

### Substudy home monitoring

The substudy dataset includes home-held measurements data collected through the patientMpower (pMp, Dublin, Ireland, www.patientmpower.com) app, installed on patients' mobile devices. For this project, we developed an algorithm for self-assessment (ASA) of symptom burden, respiratory infection surrogates, and quality of life. This includes Visual Analogue Scale (VAS) scores such as EQ5D VAS, King’s Brief Interstitial Lung Disease Health Status (K-BILD), and the Leicester Cough Questionnaire (LCQ) [30–34]. Additionally, an algorithm for spirometry and measuring oxygen saturation at rest and during exercise was integrated into the app. The data are regularly transferred to the RARE-ILD DWH using pseudonyms and kept strictly confidential, with no personally identifiable information collected [35].

## Discusssion

Registry data are extremely valuable for rare heterogeneous diseases, providing real-life insights into disease behavior and treatment response [36, 37]. Registries, unlike randomized controlled trials, operate with more flexible inclusion criteria, targeting consecutive „real world “ patients, which makes the results more affiliated with the realities of daily clinical practice [38]. The achievements of the participating centers in our previous projects were realized through trustful interaction, an integrative approach, and synergistic alignment of research plans across basic, translational, and clinical domains.

The eurILDreg approach is expected to have implications for advancing the understanding and management of ILDs, by investigating the impact of environmental factors on disease natural course and progression, determining the role of exacerbations in ILD initiation and progression, and generating a comprehensive set of big data, including deep phenome, imaginome, epigenome, proteome, transcriptome, and volatolome. Utilizing a unified digital registry platform, the analysis of this extensive dataset will employ artificial intelligence and computational modeling, advancing the understanding and management of ILDs.

The data integration allows for the identification of patterns in clinical behavior, establishing connections between clinical manifestations and underlying molecular profiles. This approach, linking clinical data to imaging, genomic information, and biomarker profiles, enhances the depth of knowledge regarding ILD. The dataset will be instrumental in the development of data-driven models leveraging machine learning algorithms. By analyzing the data comprehensively, these models may unveil previously unrecognized associations and patterns, contributing to the ongoing efforts to enhance the precision and effectiveness of ILD diagnosis. The utilization of the RARE-ILD data warehouse, coupled with advanced analytics and artificial intelligence, enables a multifaceted exploration of ILD. This approach goes beyond traditional analyses, providing a platform for uncovering intricate relationships within the data and opening avenues for the discovery of valuable insights that can shape the future of ILD research and clinical care.

Building on this collaborative experience, we advocate for our goals to be best achieved within an international network of experts, enabling the inclusion of a sufficient number of subjects to characterize the entire phenotypic spectrum across different ILDs, age groups, gather relevant biological samples for translational research, and strategically align research plans synergistically. The composition of the group reflects a selection of highly advanced and experienced clinical and basic researchers, offering a unique opportunity for cross-disciplinary collaboration and research advancements, particularly within the RARE-ILD consortium.

Through the collaborative framework provided by the consortium, we expect substantial potential for advancing future perspectives in various key areas, including the facilitation of patient-centered care. By sharing innovative tools, state-of-the-art techniques, and novel ideas within this collective research effort, we can enhance not only the efficiency and optimization of our scientific endeavors but also the translation of research findings into tangible improvements in patient management. The pooling of expertise from diverse clinical and research backgrounds within the consortium allows for the integration of patient perspectives into study designs, interventions, and healthcare delivery models. The collective expertise can lead to the identification of novel biomarkers, the refinement of diagnostic methods, and the creation of more effective treatment modalities, ultimately contributing to improved patient outcomes. We believe that expanding our network will enhance the registry's diversity and the robustness of our data, ultimately benefiting ILD research and patient care.

## Data Availability

No datasets were generated or analysed during the current study.

## Abbreviations

1STST: One-minute Sit-to-Stand Test
6MWT: Six-minute Walking Test
AKEK: Germ. Arbeitskreis der Ethik-Kommissionen, Working Group of the Ethics Committees
ASA-ILD: Algorithm for Self-Assessment in Interstitial Lung Diseases
ArtiQ: Artificial intelligence software used for real-time analysis of spirometry maneuvers
AZ: Aktenzeichen (File number)
BALF: Bronchoalveolar Lavage Fluid
BIMS: Biobank Information and Management System
CCL18: Chemokine (C–C motif) ligand 18
ChILD-EU: European Union registry for pediatric ILDs
COP: Cryptogenic organizing pneumonia
CVD-ILD: ILDs associated with collagen/vascular diseases
DIP: Desquamative interstitial pneumonia
DRKS: German Clinical Trials Register
DWH: Data Warehouse
EBC: Exhaled Breath Condensate
EDC: Electronic Data Capture
EDTA: Ethylenediaminetetraacetic Acid (a type of anticoagulant used in blood sample collection)
eNose: Electronic Nose
EQ5D VAS: EuroQol 5-Dimension Visual Analog Scale
ERS/ATS: European Respiratory Society/American Thoracic Society
eurILDreg: European Registry and Biobank for Interstitial Lung Diseases
eurIPFreg: European Registry and Biobank for Idiopathic Pulmonary Fibrosis
eurIPFnet: European IPF network
fNSIP: Fibrotic Non-Specific Interstitial Pneumonia
FVC: Forced Vital Capacity
GDPR: General Data Protection Regulation
HP: Hypersensitivity pneumonitis
HRCT: High-Resolution Computed Tomography
HRQoL: Health-Related Quality of Life
IC: Informed consent
IIPs: Idiopathic Interstitial Pneumonias
ILD: Interstitial Lung Diseases
IPF: Idiopathic Pulmonary Fibrosis
K-BILD: King's Brief Interstitial Lung Disease Questionnaire
KL-6: Krebs von den Lungen-6
LAM: Lymphangioleiomyomatosis
LCQ: Leicester Cough Questionnaire
MDD: Multidisciplinary Discussion
MIR: Medical International Research (manufacturer of Spirobank Smart)
miR-21: MicroRNA-21
miR-155: MicroRNA-155
MMP-7: Matrix Metalloproteinase-7
NIHR: National Institute of Health Research
Nonin Medical, Inc: Manufacturer of the pulse oximeter used in the study
patientMpower: Developer of the smartphone application used for monitoring
PPF: Progressive Pulmonary Fibrosis
PPFE: Pleuropulmonary Fibroelastosis
RARE-ILD: Raising Diagnostic Accuracy and Therapeutic Perspectives with the European Registry for ILD
RB-ILD: Respiratory Bronchiolitis–ILD
RCTs: Randomized Clinical Trials
SOP: Standard Operating Procedure
SP-A: Surfactant Protein A
SP-D: Surfactant Protein D
TERT: Telomerase Reverse Transcriptase
TLS: Transport Layer Security
uILD: Unclassifiable Interstitial Lung Disease
VOC: Volatile Organic Compounds
